# Supplementary material for: Empirical methods for controlling false positives and estimating confidence in ChIP-Seq peaks
Source: BMC Bioinformatics. 2008 Dec 5;9:523. doi: 10.1186/1471-2105-9-523 (PMC2628906; doi:10.1186/1471-2105-9-523)
Supplement: Additional File 1 — A variety of html documents from the USeq web site detailing the available applications, their best usage, output file type descriptions, command line menus, etc. [file 1471-2105-9-523-S1.zip › USeqUserGuides/JavaDoc.zip!/JavaDoc/util/bio/annotation/class-use/GeneGroup.html]

Uses of Class util.bio.annotation.GeneGroup


|  |  |  |
| --- | --- | --- |
| **Overview** \| **Package** \| **Class** \| **Use** \| **Tree** \| **Deprecated** \| **Index** \| **Help** | |  |
| PREV   NEXT | **FRAMES**    **NO FRAMES**     **All Classes** |  |


---


## **Uses of Class util.bio.annotation.GeneGroup**

| Packages that use GeneGroup | |
| --- | --- |
| **trans.anno** | For associating intervals, binding regions, and binding peaks with GFF annotation. |
| **trans.roc** | For manipulating the BAC dmel spike in data and generating ROC curves. |
| **util.bio.annotation** | For representing gene annotation. |
| **util.bio.parsers.gff** | GFF2 and GFF3 parsers. |

Uses of GeneGroup in trans.anno

| Methods in trans.anno that return GeneGroup | |
| --- | --- |
| `static GeneGroup[]` | `AnnotateRegions.filterGeneGroups(GeneGroup[] genes, java.util.HashSet cgNames)`             Returns an array of GeneGroup whose names were found in the Hash |
| `GeneGroup` | `BindingRegion.getGeneGrp3Prime()` |
| `GeneGroup` | `BindingRegion.getGeneGrp5Prime()` |

| Methods in trans.anno with parameters of type GeneGroup | |
| --- | --- |
| `void` | `BindingRegion.compare(GeneGroup geneGrp)`             Compares a binding region to a gene grp looking for where it is in relation to the binding region, 5', 3', overlap, contained. |
| `static void` | `AnnotateRegionsWithGeneList.compareBindingRegionsVsGeneGrps(GeneGroup[] geneGroups, BindingRegion[] bindingRegions)`             Does a complete scan, could be optimized. |
| `static void` | `AnnotateRegions.compareBindingRegionsVsGeneGrps(GeneGroup[] geneGroups, BindingRegion[] bindingRegions)`             Does a complete scan, could be optimized. |
| `static GeneGroup[]` | `AnnotateRegions.filterGeneGroups(GeneGroup[] genes, java.util.HashSet cgNames)`             Returns an array of GeneGroup whose names were found in the Hash |
| `int` | `AnnotateRegionsWithGeneList.findDistanceToATG(BindingRegion br, GeneGroup gp)`             Finds the distance to conservative estimate of an ATG, returns 0 if overlaps. |
| `int` | `AnnotateRegions.findDistanceToATG(BindingRegion br, GeneGroup gp)`             Finds the distance to conservative estimate of an ATG, returns 0 if overlaps. |
| `int` | `AnnotateRegionsWithGeneList.findDistToClosestTranscript(BindingRegion br, GeneGroup gp)`             Finds the distance to conservative estimate of start of first exon, returns 0 if overlaps. |
| `int` | `AnnotateRegions.findDistToClosestTranscript(BindingRegion br, GeneGroup gp)`             Finds the distance to conservative estimate of start of first exon, returns 0 if overlaps. |

Uses of GeneGroup in trans.roc

| Methods in trans.roc that return GeneGroup | |
| --- | --- |
| `static GeneGroup[]` | `ParsePatternedWindows.extractGeneGroups(java.util.HashMap geneGroups, java.lang.String[] geneList)`             Pulls out a sub set of GeneGroups with the names in the geneList. |

| Methods in trans.roc with parameters of type GeneGroup | |
| --- | --- |
| `static RocWindow[]` | `ParsePatternedWindows.fetchProximalWindows(GeneGroup[] geneGrps, RocWindow[] windows, int sizeUpstream)` |

Uses of GeneGroup in util.bio.annotation

| Methods in util.bio.annotation that return GeneGroup | |
| --- | --- |
| `GeneGroup` | `GeneRep.getGeneGrp()` |

Constructors in util.bio.annotation with parameters of type GeneGroup

`GeneRep(TransGroup[] transGrps,
GeneGroup geneGrpRef)`

Uses of GeneGroup in util.bio.parsers.gff

| Methods in util.bio.parsers.gff that return GeneGroup | |
| --- | --- |
| `GeneGroup[]` | `DmelRel4Extractor.getGeneGroups()`             Final product from the extractor. |

---


|  |  |  |
| --- | --- | --- |
| **Overview** \| **Package** \| **Class** \| **Use** \| **Tree** \| **Deprecated** \| **Index** \| **Help** | |  |
| PREV   NEXT | **FRAMES**    **NO FRAMES**     **All Classes** |  |


---
